# The Cost of Antibiotic Mass Drug Administration for Trachoma Control in a Remote Area of South Sudan

**DOI:** 10.1371/journal.pntd.0001362

**Published:** 2011-10-11

**Authors:** Jan H. Kolaczinski, Emily Robinson, Timothy P. Finn

**Affiliations:** 1 Malaria Consortium – Africa Regional Office, Kampala, Uganda; 2 London School of Hygiene and Tropical Medicine, London, United Kingdom; 3 Malaria Consortium – South Sudan Country Office, Juba, South Sudan; Yale School of Medicine, United States of America

## Abstract

**Background:**

Mass drug administration (MDA) of antibiotics is a key component of the so-called “SAFE” strategy for trachoma control, while MDA of anthelminthics provides the cornerstone for control of a number of other neglected tropical diseases (NTDs). Simultaneous delivery of two or more of these drugs, renowned as “integrated NTD control,” is being promoted to reduce costs and expand intervention coverage. A cost analysis was conducted alongside an MDA campaign in a remote trachoma endemic area, to inform budgeting for NTD control in South Sudan.

**Methods and Findings:**

A first round of antibiotic MDA was conducted in the highly trachoma endemic county of Mayom, Unity state, from June to August 2010. A core team of seven staff delivered the intervention, including recruitment and training of 44 supervisors and 542 community drug distributors. Using an ingredients approach, financial and economic costs were captured from the provider perspective in a detailed costing database. Overall, 123,760 individuals were treated for trachoma, resulting in an estimated treatment coverage of 94%. The economic cost per person treated was USD 1.53, excluding the cost of the antibiotic azithromycin. Ninety four per cent of the delivery costs were recurrent costs, with personnel and travel/transport costs taking up the largest share.

**Conclusions:**

In a remote setting and for the initial round, MDA of antibiotics was considerably more expensive than USD 0.5 per person treated, an estimate frequently quoted to advocate for integrated NTD control. Drug delivery costs in South Sudan are unlikely to decrease substantially during subsequent MDA rounds, as the major cost drivers were recurrent costs. MDA campaigns for delivery of one or more drugs in South Sudan should thus be budgeted at around USD 1.5 per person treated, at least until further costing data for delivery of other NTD drugs, singly or in combination, are available.

## Author Summary

Trachoma is one of a group of so-called “neglected tropical diseases” (NTDs) for which safe and effective treatments are available. The International Trachoma Initiative oversees donation of the antibiotic azithromycin to endemic countries. Delivery of this drug to communities affected by trachoma is the responsibility of national programmes and their implementing partners, and should be conducted as part of a comprehensive control strategy termed “SAFE,” which includes trichiasis surgery, health education and water/sanitation interventions. There are little data on how much the different components of a trachoma control programme cost and none from South Sudan. To inform budgeting to scale up control of trachoma, and of other NTDs whose control relies on large-scale mass drug administration (MDA), the present study set out to determine the cost per person treated when antibiotics were delivered through a vertical campaign that covered 94% of the target population in a remote trachoma endemic area of South Sudan. The average economic cost per person treated was USD 1.53, which included all inputs not paid for in cash except for the cost of the donated azithromycin and the opportunity cost of community members attending treatment.

## Introduction

Since 2007, financial support for the control or elimination of some key neglected tropical diseases (NTDs) has increase substantially [1]–[4]. The NTDs largely benefitting from this attention are onchocerciasis, lymphatic filariasis (LF), soil-transmitted helminth infections, schistosomiasis and trachoma. The rationale for focusing on this group of diseases is based on the fact that safe and effective preventive chemotherapy (PCT) options are available for all of them, either free of charge or at low cost, and on the assumption that previously separate distributions of these drugs by stand-alone vertical programmes could be easily combined under one structure or become part of other large-scale distributions of public health commodities such as insecticide-treated mosquito nets, hence substantially reducing delivery costs [5]. A figure of around USD 0.5 per person treated per year for co-administration of PCT has been extensively used for advocacy [6]–[8], while there is limited empirical evidence supporting this estimate [9]–[11]. For programmatic purposes, particularly to budget for scale up of PCT delivery to previously untreated areas, there is thus a need to collect accurate cost data. Furthermore there is a need to develop systems for routine collection of NTD programme cost data to determine whether economies of scale and/or scope exist and to estimate the cost-effectiveness and cost-benefit of stand-alone and ‘integrated’ NTD control programmes [12].

Investigations into the cost and cost-effectiveness of PCT delivery for the above diseases have been undertaken to varying degrees, depending on the availability of data and the principal interest of the researchers undertaking the analyses. Delivery costs for large-scale deworming through schools in Kenya, for example, have been estimated at USD 0.23 per child treated with albendazole or USD 0.95 for combined albendazole and praziquantel treatment (both estimates including the cost of the drug) [13]. Estimates of delivery costs for mass drug administration (MDA) of anthelminthics from other countries have ranged from USD 0.03 to USD 0.54 [10], [13], [14], [15]. Similar cost estimates have been arrived at for MDA campaigns aimed at LF elimination, although the upper bound was significantly higher at USD 2.23 for the per capita cost of Haiti's first MDA round [12]. As frequently encountered with economic analyses, these results are not necessarily readily comparable, as investigators choose to use different costing perspectives and approaches [16]. The results generated may thus not be suitable for budgeting purposes.

For trachoma, a number of cost and cost-effectiveness studies have been conducted [17]–[22] and detailed guidance on how to conduct a cost-effectiveness analysis for this disease has been provided [23]. The present study aimed to build on this evidence by generating critical information on the per-capita cost of implementing a MDA campaign for trachoma. The intervention was supported by the United States Agency for International Development Neglected Tropical Disease (NTD) Control Program and delivered in a remote trachoma endemic area of South Sudan that had not benefited from intervention. Cost data specific to this setting was required to predict the likely cost of repeating this exercise in the same area, scaling up antibiotic distribution to other trachoma endemic areas of the country and to provide a general estimate of how much it might cost to deliver one or more drugs for NTD control through campaigns in this post-conflict setting.

## Methods

### Ethical Considerations

The MDA campaign was conducted on behalf of the Ministry of Health, Government of South Sudan (MoH-GoSS), and in close collaboration with state and county MoH representatives. Implementation followed treatment guidelines provided by ITI and WHO [24], [25]. The costing study only used data on expenditures and non-financial inputs incurred by Malaria Consortium in the implementation of the campaign. Collection of these data did not involve human subjects and therefore did not require ethical approval.

### MDA Description

The overall purpose of the MDA campaign was to deliver antibiotics to the highest possible number of individuals eligible for treatment in an area of South Sudan shown to have prevalences of 57.5% and 39.8%, respectively, of trachomatous inflammation-follicular and trachomatous inflammation-intense in children aged 1–9 years. In the same area, the prevalence of trachomatous trichiasis in adults was found to be 19.2% [26].

The International Trachoma Initiative (ITI) recommends at least 90% of the population should be treated with antibiotics to lower the risk of recurrent infection [24]. Children below six months of age should be treated with tetracycline eye ointment for six weeks (by their parents or care taker), while older children and adults should receive a single dose of azithromycin. To minimize the risk of choking on tablets, children age six months to five years are generally provides with a paediatric oral suspension (POS) formulation of azithromycin, while older children and adults receive the drug in tablet form. For both formulations the dose was determined according to height by means of a gradated dose pole, as recommended by ITI and WHO [24], [25].

Between June and August 2010, 417 villages in all ten payams of Mayom County, Unity State, received antibiotics (azithromycin and tetracycline) through a MDA campaign. South Sudan has a four-tier administrative structure comprised of states (1^st^), counties (2^nd^), payams (3^rd^) and bomas (4^th^). The rainy season in this part of the country starts in about May, which unfortunately meant that the majority of fieldwork had to be conducted during the worst season for implementation. Ideally, implementation would have been conducted during the dry season, but in the present case this was not feasible due to various logistical and financial delays.

A core team led by a MDA coordinator and consisting of six training and treatment supervisors (TATS) conducted the campaign. A Malaria Consortium field officer and a logistician also supported the team at various stages of the MDA, amounting to a total of 30 and 22 days, respectively. In addition, 542 community drug distributors (CDDs) and 44 supervisors were recruited and trained in the process; both of these staff categories were paid an incentive, while the core team and their support staff were on full salaries.

The number of CDDs required to treat each boma was calculated using population data from the 2008 national census. Approximately one CDD was recruited for every 40 households or 240 people (as the average household size was estimated to be six people). Two CDDs were paired up for the duration of the campaign to ensure that at least one from each pair lived in the area to be treated and that at least one was literate. Each CDD had to attend a three-day training session to acquire a basic background on trachoma and its treatment, and be informed of his/her role in the MDA. The CDD's role was to conduct a census of their allocated households, pick up drugs from an agreed location, return to and correctly treat their allocated households, and return equipment, drugs and the census and treatment registers to a Malaria Consortium staff member. Each CDD was provided with a set of equipment at the end of the training: T-shirt, ID card, backpack, pens/pencils/erasers, notebook, clipboard, plastic folder, census and treatment register, and spray paint/stickers to mark houses that had been treated. Each pair of CDDs was also given a trachoma flip chart to facilitate health education sessions, a wooden dose pole to determine the amount of azithromycin to be given to each individual, a cup, and water purification tablets to treat all drinking water before it was given out to facilitate swallowing of azithromycin tablets. Supervisors were provided with the same equipment as CDDs, as well as with a pair of gumboots and a raincoat. They were also loaned a bicycle for the duration of the MDA in their payam (usually around ten days).

Two Land Cruiser HZJ Troop Carriers (Toyota Motor Corporation, Toyota City, Aichi, Japan) owned by Malaria Consortium and two drivers were allocated to the MDA campaign for its entire duration. Each of the vehicles was equipped with a high frequency radio (Codan Ltd., Adelaide, Australia), a handheld satellite phone (Thuraya, Abu Dhabi, United Arab Emirates) and an in-car charger, to allow communication between teams and with the field office at all times. A third vehicle and driver were hired for one month during the campaign, but it lacked above equipment and was not suitable for off-road driving.

The MDA campaign consisted of three distinct implementation phases: a start-up phase amounting to a total of 57 days during which health education and training materials were developed, equipment was purchased and transported to the state capital Bentiu, TATS were recruited and trained, and local authorities were made aware of the upcoming MDA and its purpose. After the start-up phase was complete, MDA delivery was conducted payam-by-payam using approximately the same procedure and timeframe in each and amounting to a total of 88 days, 27 of which were spent on training CDDs. The procedure applied consisted of CDD and supervisor training (2–3 days), a census in each village (2 days), drug pick-up (1 day), MDA delivery (2 days), and drop-off of drugs and treatment registers (1 day). The MDA was conducted using central point or house-to-house delivery, as agreed upon by the CDDs and the community during the census. Once all payams had been treated, a closure phase of 24 days was required during which equipment was transported and stored, inventories were updated, data were entered and analysed, and a report was prepared.

### Collection of Cost Data

A detailed costing database was developed using an ingredients approach to capture financial and economic costs as incurred by Malaria Consortium. The costing template and methodology described in an earlier costing study on NTD mapping in South Sudan were used for this purpose [27]. Some costs that could not be captured during the fieldwork were subsequently estimated from financial expenditure records. Average exchange rates for the period 1 May to 31 August 2010 were used for currency conversions, as provided by OANDA (http://www.oanda.com/currency/historical-rates). The rates were: 1 United States Dollars (USD) = 2.26 Sudanese Pound (SDG) or 1 USD = 2,197 Ugandan Shillings (UGX).

Both financial and economic costs were estimated from the perspective of the provider [28], in this case Malaria Consortium. The only input not captured using this approach was the time of community members required to attend sensitization and treatment sessions. In addition, we chose not to include the cost of azithromycin, as ITI will donate and deliver this drug to trachoma endemic countries for the foreseeable future. Furthermore, inclusion of the economic cost of this drug, estimated at USD 278 per bottle of 30 tablets and USD 39 per 1,200 mg bottle of POS (ITI, pers. com.) would have skewed the cost-estimate towards the drug, while we were ultimately interested in estimating a comprehensive delivery cost as incurred by the provider. The cost of tetracycline, however, was included, as this drug has to be procured by the provider to ensure that all eligible age groups can be provided with trachoma treatment.

Financial costs captured were the cash expenditures made to enable implementation of the campaign. For capital items, these were estimated for the total number of days they were required to organize and implement the MDA campaign using straight-line depreciation, followed by calculation of an average financial daily cost. Economic costs captured the value of all resources required for the campaign, including opportunity costs of equipment that was used but not paid for and a proportion of the costs of capital items with a value over USD 100 and an expected useful life of more than one year that were used in the campaign [29]. The time of CDDs was not included as an economic cost, because they were paid a per-diem as compensation for their time. Capital items were discounted over their estimated useful life using the recommended discount rate of 3% [30], [31]. Daily economic costs were calculated for all capital items and multiplied by the appropriate number of days in use during the MDA campaign. Based on our experience of working in the harsh environment of South Sudan, we estimated the useful life of vehicles and high frequency radios (fitted to vehicles) to be four years and two years for all other items.

All resources used to conduct the costing study were excluded from the analysis. Training costs, however, which could potentially be incurred only once if knowledge and staff were retained over subsequent MDA rounds, were included as an integral part of the campaign costing. This was felt to be necessary, because the study aimed to estimate the cost of scaling up MDA to the substantial trachoma endemic areas of South Sudan that have not been targeted with interventions to date [32], hence incurring these training costs, but also because high attrition of community volunteers [11] is likely to results in the same or similar training needs for subsequent MDA rounds in the same geographic area.

To be consistent with our previous costing study in South Sudan [27] we applied an overhead of 25% to the financial cost estimates for all budget lines. In our experience this estimate provides an accurate reflection of overheads associated with implementation in this post-conflict setting.

### Outcomes

The outcome that the present analysis aimed to estimate was the cost per person treated through an MDA campaign, regardless of the type of antibiotic treatment or its formulation. Given that high coverage with azithromycin and tetracycline is required for effective control, we decided that there was no reason to investigate potential differences in delivery costs between antibiotic types or formulations.

### Sensitivity Analyses

One-way sensitivity analyses were conducted to explore the effects of key assumptions on the cost estimate. The effects of varying the following parameters were explored: i) the discount rate was reduced to 0% or increased to 10%; ii) the assumed lifespan of vehicles was increased to 7.5 years, and iii) the exchange rate was modified from the average of SDG 2.26 per USD provided by OANDA to an average of SDG 2.45 per USD, as used by the KCB bank in Juba over the implementation period.

## Results

The total financial cost of the campaign amounted to USD 169,084 including a 25% overhead (Table 1). The major cost drivers were recurrent rather than capital costs, led by personnel (41.3%) and followed by travel/transport (29.1%) and accommodation/sustenance (10.0%). The largest proportion of financial costs was incurred during the actual MDA delivery period (86.6%), while the start up and closure phases only required moderate investment (Table 2). The total economic cost was USD 189,889, including the same overhead as that included in the financial cost (Table 1). Economic costs were slightly higher than financial costs, because this estimate captured non-financial contributions to the implementation of the campaign. The difference between these two estimates was greatest for the categories of accommodation/sustenance and MDA/IEC consumables and other charges (Table 1), largely because the team was able to stay free of charge with other non-governmental organizations during part of the campaign and because the state/county administrations provided the training facilities. In other settings, even within Unity state, these contributions would have to be paid for, which is why they were included as part of the economic costs.

**Table 1:** Financial and economic costs, and associated cost profiles, for antibiotic MDA in Mayom County.

	Total financial cost (USD)	% of financial cost*	Total economic cost (USD)	% of economic cost*
**Capital costs**				
Vehicles	5,369	4.0%	5,718	3.7%
Communication & IT equipment	558	0.4%	601	0.4%
Accommodation equipment	446	0.3%	467	0.3%
MDA & IEC equipment	2,348	1.7%	2,469	1.6%
**Subtotal**	**8,721**	**6.4%**	**9,255**	**5.9%**
**Recurrent costs**				
Travel & transport	39,306	29.1%	39,465	25.3%
Vehicle fuel & maintenance	3,384	2.5%	3,384	2.2%
Accommodation & sustenance	13,513	10.0%	22,479	14.4%
MDA & IEC consumables & other charges	8,063	6.0%	17,449	11.2%
Drugs (tetracycline/azithromycin)	5,116	3.8%	5,116	3.3%
Communication	1,246	0.9%	1,246	0.8%
Personnel	55,919	41.3%	57,679	37.0%
**Subtotal**	**126,546**	**93.6%**	**146,817**	**94.1%**
**Subtotal**	**135,267**		**156,072**	
Overhead (25%)	33,871		33,817**	
**Total**	**169,084**		**189,889**	
**Number of persons treated**	**123,760**		**123,760**	
**Cost per person treated**	**1.37**		**1.53**	

*Proportions were calculated on the subtotal, i.e. excluding the overhead.

**Overhead as calculated for financial costs.

**Table 2:** Financial and economic costs, and associated cost profiles, for the three phases of antibiotic MDA.

	Total financial cost (USD)	% of financial cost*	Total economic cost (USD)	% of economic cost*
**Campaign phases**				
Start-up phase	13,470	10.0%	18,907	12.1%
MDA delivery	117,086	86.6%	129,784	83.2%
Closure phase	4,711	3.5%	7,381	4.7%
**Subtotal**	**135,267**		**156,072**	
Overhead (25%)	33,817		33,817**	
**Total**	**169,084**		**189,889**	

*Proportions were calculated on the subtotal, i.e. excluding the overhead.

**Overhead as calculated for financial costs.

The MDA campaign delivered antibiotic treatments to a total of 123,760 individuals in Mayom county, which translated into 94% coverage of the estimated population. For the first time ever this area benefitted from at least one of the component of the SAFE strategy for trachoma control. The average economic cost per person treated was USD 1.53, excluding the cost of azithromycin. If the economic cost of the donated antibiotic had been included, the estimated economic cost per person treated would have been USD 34.2 (data not shown).

Varying the underlying assumptions had limited effect on the estimated economic cost per person treated (Table 3). Most pronounced was the use of the exchange rate applied by the main bank in Juba, as opposed to the figure provided by OANDA, reducing the cost per person treated to USD 1.48.

**Table 3:** Sensitivity of economic cost estimate to underlying assumptions.

Assumptions tested	USD/person treated	% deviation from base case
Base case	1.53	-
Discount rate:		
0%	1.53	0%
10%	1.55	+1.3%
Vehicle lifespan:		
7.5 years	1.51	−1.3%
Exchange rate (SDG/USD):		
2.45	1.48	−3.3%

## Discussion

The aim of the present analysis was to estimate the cost of delivering one, or possibly more, drug(s) through MDA campaigns in South Sudan, thus informing budgeting for control of trachoma and other key NTDs in the country. The costing study established that a first round of MDA in the remote area of Mayom county incurred an economic cost of USD 1.53 per person treated. This estimate was considerably higher than that of USD 0.5, frequently quoted as the approximate annual per capita cost for simultaneous delivery of multiple drugs to control some key NTDs [6]–[8]. This substantial difference between the two cost estimates can largely be explained by the fact that the latter figure is not based on in-depth costing work for a specific setting.

In the present case, the setting was one of the hardest to reach, and hence most costly implementation areas in Sub-Saharan Africa. Moreover, logistical and financial delays meant that implementation had to be conducted during the rainy season, slowing down fieldwork. In addition, this was the first MDA round for trachoma control in Mayom county, necessitating a start-up period to develop the training and health education materials, develop a baseline list of villages, which did not exist previously, and develop the most practical MDA implementation methodology. The cost estimate may therefore be considered a ‘worst-case scenario’, rather than a figure that is generally applicable for large-scale PCT (co-)administration in other parts of Sub-Saharan Africa, although this remains to be confirmed. Our estimate would certainly be applicable to the delivery of first rounds of antibiotics or other PCT for NTD control in most areas of South Sudan, as the remoteness and operational constraints of Mayom are the rule rather than an exception in this country.

Unfortunately it is hard to judge how our estimate compares to other settings, as we were unable to identify any other estimates of the delivery cost for trachoma MDA that were directly comparable to the approach used here. The seemingly most comparable result was obtained as part of a comparison of different treatment strategies in Mali [21]. Costs were, however, not directly estimated during the study but extrapolated from data provided by the Malian National Programme for Prevention of Blindness for antibiotic distribution at district level. Assuming the drug was free of charge and including an estimate of the societal cost associated with attending the distribution, the study arrived at an estimated cost per person treated of USD 0.25 for mass treatment of all residence [21]. It is unclear whether this estimate was derived using the same ingredients as in the present study. One ingredient that was clearly not included in the analysis from South Sudan was the opportunity cost of the people attending treatment. Two methods – central point or house-to-house distribution – were used in Mayom depending on the communities' decision, but no data were collected on which approach was used where. It was therefore not feasible to include the correct economic cost associated with community members attending distribution, or to establish whether there may have been a difference in cost between the two distribution methods.

Drug delivery costs in South Sudan would likely be slightly lower in areas nearer the capital Juba, as this would reduce the high transport costs. In the present case, supplies and equipment had to be brought from Juba to Unity, a journey taking at least three days by truck. Implementation in areas near Juba may also be conducted over a shorter timeframe, because accessibility of communities is generally better and a higher proportion of the community is literate. Furthermore, the population density near the capital is higher, meaning that it may be feasible to treat more people with similar inputs, for example by utilizing more central point than house-to-house distribution. This could increase the denominator and hence decrease the cost per person treated. Costs may also decrease when the materials and experience developed during the initial campaign are applied in subsequent rounds. Further cost analysis by implementing partners is clearly required to answer these questions and assist in future budgeting.

Given our experience of operating in South Sudan, we nevertheless feel that implementation in areas other than Mayom county and the delivery of subsequent, rather than initial, MDA rounds are unlikely to lead to significant cost savings. Most trachoma endemic areas of South Sudan are remote, with households dispersedly located, and hence comparable to Mayom. Staff turnover including that of CDDs is high [11], resulting in loss of institutional memory and requiring a large amount of costly re-training from year to year. In the present MDA round there was also no obvious opportunity to increase the scale of the intervention with existing resources, as the core MDA team had no spare capacity to allow coverage of additional areas. Scaling up geographical coverage while maintaining quality, safety and at least 90% population coverage would thus be associated with hiring more personnel and using more transport resources, both of which drove the overall implementation cost in the Mayom campaign and would lead to a similar delivery cost per person treated if the same approach was scaled up. For subsequent MDA campaigns in Mayom, as well as for the scale up of trachoma control to all other counties of Unity state, it therefore seems prudent to budget an average cost of USD 1.5 per person treated, unless obvious cost savings are identified during repeated rounds. Accordingly, the estimated cost of treating the population in all nine trachoma endemic counties of Unity State would exceed USD 1 million annually and require a yearly donation of azithromycin valued at USD 16 million.

Further costing work on drug delivery through community-based distribution mechanisms in South Sudan and elsewhere would be useful, particularly with regards to estimating the actual cost and cost-effectiveness of co-administering PCT in areas endemic for more than one NTD [33]. In the present case, the intervention area is also endemic for schistosomiasis (Malaria Consortium, unpublished) and is being targeted by a programme for integrated community-case management (iCCM) to treat malaria, pneumonia and diarrhoea [34], [35]. While drug safety recommendations would currently not allow the co-administration of antibiotics with praziquantel for schistosomiasis control [25], there may be ways in which iCCM and its support/supervision system could be harnessed to contribute to trachoma and/or schistosomiasis control. There was some collaboration between the trachoma and iCCM intervention during this first MDA round, but both interventions had only just started and were largely concerned with achieving their own objectives rather than exploring opportunities for integration. Now that iCCM has been fully established it would be beneficial to estimate what additional resources are required to deliver MDAs for trachoma and schistosomiasis control as part of this structure rather than through a vertical campaign, and whether this is likely to be as effective but cheaper. Similarly, further experience and cost data is urgently required to determine how South Sudan could establish an innovative platform for community-based delivery of a broad range of public health interventions; one of the opportunities offered by this post-conflict setting that has not been taken up [11].

### Conclusion

In a remote setting and for the initial round, the delivery of antibiotics for trachoma control through an MDA campaign was three times as expensive as the figure commonly used in international advocacy for simultaneous delivery of a package of drugs to prevent or treat multiple NTD. Costs for delivering one or more drug(s) through MDA campaigns in South Sudan are unlikely to decrease substantially during subsequent rounds, as the major cost drivers were recurrent costs and unlikely to decline dramatically. Until further cost data are generated, MDA campaigns in South Sudan should thus be budgeted at a delivery cost of about USD 1.5 per person targeted.
